# Editorial Outline: Overview of Recent Developments in Biobased and Biodegradable Polymers

**DOI:** 10.3390/polym17243264

**Published:** 2025-12-09

**Authors:** Evangelia Balla, Dimitrios N. Bikiaris

**Affiliations:** Laboratory of Polymer Chemistry and Technology, Department of Chemistry, Aristotle University of Thessaloniki, 54124 Thessaloniki, Greece; evampadio@chem.auth.gr

## 1. Introduction

The reliance on fossil-based materials that are low-cost, easy to produce, and lightweight has led to growing environmental challenges. In response, global concerns for sustainability have promoted a shift toward biobased polymers, a class of substances entirely or partially derived from biomass or biological products including microbial, plant, and animal sources [1]. The choice of appropriate feedstock should take into account both the micro- and macroscopic polymer characteristics, including biodegradability assessment and a full life cycle assessment (LCA) of the new polymers [2]. The properties of biobased polymers such as recyclability, low density, minimal health risks, potential biodegradability, toughness, and good thermal stability make them useful for diverse industrial applications, particularly in packaging, biomedical, and consumer products. Over recent years, the biobased plastic production capacity has been steadily growing, with an estimated compound annual growth rate of ∼14 % by 2027 [3].

The significance of biobased polymers lies in their potential to mitigate pollution and reduce dependence on limited fossil resources while lowering CO_2_ emissions by up to 70% compared to conventional polymers [4]. Consequently, extensive research efforts are now focused on optimizing their synthesis, processing, and large-scale use as viable replacements for conventional plastics [1]. The degradation mechanisms of biobased polymers, their biocompatibility, and their mechanical and thermal characteristic have been extensively studied and reported in various review articles [5].

**Figure 1:**
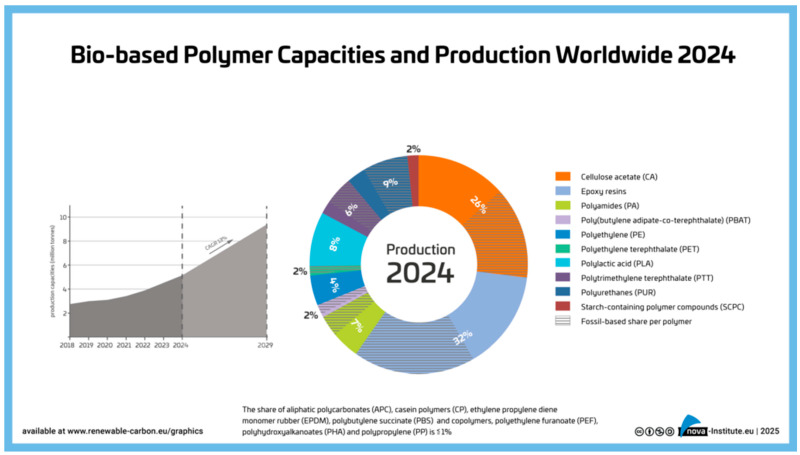
Biobased polymer capacities and production worldwide, 2024 [7].

In 2024, the global production of biobased polymers reached 4.2 million tons. Major industries that use biodegradable biobased polymers include pulp and paper, agriculture, beverages, and medical supply manufacturers [6]. Among the most commonly used biobased polymers, cellulose acetate and epoxy resins dominated the market, representing 26% and 32% of total biobased polymer production, respectively. They were followed by polyurethanes (PU), poly(lactic acid) (PLA), polyamides (PA), polyethylene (PE), poly(trimethylene terephthalate) (PTT), and poly(ethylene terephthalate) (PET). Other categories of biobased polymers used widely in 2024 are illustrated in Figure 1. However, the main drawback to most of these is that they behave in the environment in the same way as fossil-based polymers. They are non-degradable polymers and are continuing to contaminate the environment.

There has also been remarkable progress in both biobased and biodegradable materials in this field. This Editorial summarizes recent work related to the sector and provides a brief overview of recent developments. It also highlights the most important findings of each article.

## 2. Key Research Themes and Developments

### 2.1. Biobased Polymers for Environmental and Circular Applications

The growing role of biobased polymers in supporting sustainability and the circular economy has been highlighted through recent advances in the field. Apicella et al. [8] examined the degradation of PLA and PBS-based films in aquatic environments, showing that warm, light conditions accelerate the formation of bio-microplastics, providing key insights for improving environmental performance. Similarly, Jamnongkan et al. [9] discuss the essential role of bioplastics in achieving EU circular economy goals, emphasizing both the potential and the challenges of large-scale commercialization. Fabrizio Olivito et al. [10] developed biobased polyurethane foams with excellent sorption capacities for diesel and gasoline, high reusability over multiple cycles, and promising scalability for industrial water purification. Bioplastics are pivotal in global efforts to reduce CO_2_ emissions and fossil resource utilization. Terzopoulou et al. [3] discuss their concerns about the environmental impact of bioplastics, which remains unclear, including issues around raw material sourcing, land use effects, competition with food production, end-of-life management, and recyclability. Finally, they express their concerns in terms of regulatory development.

### 2.2. Advances in Biomedical and Specialized Applications

Biobased polymers offer an appealing alternative to traditional petroleum-based plastics, exhibiting comparable or even superior properties while significantly reducing environmental impacts. Their compatibility with various applications ranges from packaging to biomedical devices and specialized applications [11]. Terzopoulou et al. [12] provide a comprehensive review of PLA copolymers, highlighting a decade of innovation in the developed synthetic strategies, enhancement of desired properties, and diverse applications ranging from packaging to biomedicine. Their work shows how structural tailoring of PLA can balance both biodegradability and mechanical performance. Tang et al. [13] investigated the surface modification of nano-hydroxyapatite/polymer composites for bone tissue repair applications. They demonstrate how bioactive polymer matrices can improve mechanical stability and interfacial compatibility in biomedical devices. Finally, Bikiaris et al. [14] reviewed the presence of microplastics in cosmetic and personal care products, emphasizing the urgent need for sustainable, biobased, and biodegradable polymer alternatives to replace conventional microbeads.

### 2.3. Sustainable Extraction, Processing, and Green Chemistry Approaches

The transition toward greener polymer production relies heavily on sustainable extraction methods and eco-friendly synthesis routes. The adoption of biobased polymers represents a crucial step in eliminating plastic pollution and fostering a greener economy [11]. In this context, Magalhães et al. [15] present an overview of environmentally friendly techniques for the extraction and modification of cellulose, emphasizing low-energy and solvent-free processes that minimize environmental impact. Similarly, Mattiello [16] et al. focused on keratin recovery from both wool and chicken feathers using refined chemical extraction. The authors demonstrated how agricultural and industrial waste can be transformed into valuable biopolymers for functional applications. Complementing these efforts, Balla et al. [17] reviewed the development of non-isocyanate polyurethanes (NIPUs) as a substitute to traditional polyurethanes. In their work, they summarize NIPU synthetic pathways, recyclability, and potential as safer, sustainable alternatives to conventional polyurethanes.

### 2.4. Food Waste for Use in Smart and Functional Biobased Materials

Innovations in smart and functional materials are expanding the possibilities regarding the use of biobased polymers derived from food waste byproducts in sustainable packaging and advanced applications. This concept can lead to a “closed-loop” food waste management system. For instance, Botalo et al. [18] developed UV-resistant edible coatings and films composed of alginate, whey protein, and curcumin. Their films showed enhanced hydrophobicity and antioxidant performance, while curcumin improved UV-blocking efficiency, reduced water vapor transmission, and prolonged the shelf life of coated apples. The films also exhibited reversible color changes upon ammonia exposure, highlighting their potential as smart, active packaging materials for light-sensitive foods. Complementarily, Balla et al. [19] synthesized novel poly(butylene succinate)–cutin copolymers via in situ melt polycondensation, revealing strong interactions and enhanced crystallinity, thermal stability, and biodegradability. The incorporation of natural cutin, the byproduct of tomato peels, improved material performance while maintaining environmental compatibility.

### 2.5. Smart Solutions for Food Packaging Applications

Nowadays most packaging materials are derived from fossil carbon sources, which are favored for their versatility, esthetic appeal, and cost-effectiveness [20]. Nevertheless, the growing demand for sustainable food systems has accelerated research in smart and biobased packaging technologies. As highlighted by Stoika et al. [20], innovative packaging systems integrated with intelligent and active functionalities are able to monitor and preserve food quality while minimizing waste. Smart materials incorporating natural additives, enzymes, or biopolymers can enable the real-time detection of spoilage and extend shelf life, offering a sustainable alternative to food packaging.

Lestido-Cardama et al. [21] emphasize that biobased and biodegradable elements are increasingly being used in food contact materials. The presence of unknown oligomers in their structure combined with migration risks calls for advanced analytical tools (gas chromatography–mass spectrometry (GC–MS) or liquid chromatography–mass spectrometry (LC–MS), Orbitrap) and harmonized toxicological assessment protocols to guarantee consumer safety and material integrity. Meanwhile, Kaur et al. [22] underline the technological and economic challenges of scaling up microbial-based biopolymers, pointing to the need for cost-effective, aseptic production and enhanced mechanical performance. Recent innovations (including nanocomposite reinforcement and hybrid systems) can improve properties for food preservation. With the successful scaling up of monomer production for biobased polymers analogous to conventional ones, biobased polymer applications are expected to reach an industrial level. This progress will help achieve cost competitiveness with petroleum-based counterparts [23].

In alignment with these advances, the Horizon Europe-funded GRECO project, coordinated by Aristotle University of Thessaloniki [24], is developing innovative biobased, biodegradable, and recyclable PLA copolymers for food packaging applications and functional coatings, via the use of green catalysts. The project shows how European collaboration is advancing the transition toward low-carbon and circular food packaging systems. Finally, the project aims to demonstrate the life cycle and techno-economic feasibility of greener, safer polymer value chains through a safe-and-sustainable-by-design strategy. This probably represents the future of biobased and biodegradable polymers for several applications.
